# Data on English coda voicing contrast under different prosodic conditions produced by American English speakers and Korean learners of English

**DOI:** 10.1016/j.dib.2022.108816

**Published:** 2022-12-15

**Authors:** Sahyang Kim, Jiyoun Choi, Taehong Cho

**Affiliations:** a Department of English Education, Hongik University, Seoul, Korea; b Department of Social Psychology, Sookmyung Women's University, Seoul, Korea; c Hanyang Institute for Phonetics and Cognitive Sciences of Language, Department of English Language and Literature, Hanyang University, Seoul, Korea

**Keywords:** English coda voicing contrast, L1 english, L2 english, Korean learners, Focus, Vowel duration, Formants

## Abstract

This data article provides acoustic data for individual speakers’ production of coda voicing contrast between stops in English, which are based on laboratory speech recorded by twelve native speakers of American English and twenty-four Korean learners of English. There were four pairs of English monosyllabic target words with voicing contrast in the coda position (*bet-bed, pet-ped, bat-bad, pat-pad*). The words were produced in carrier sentences in which they were placed in two different prosodic boundary conditions (Intonational Phrase initial and Intonation Phrase medial), two pitch accent conditions (nuclear-pitch accented and unaccented), and three focus conditions (lexical focus, phonological focus and no focus). The raw acoustic measurement values that are included in a CSV-formated file are F0, F1, F2 and duration of each vowel preceding a coda consonant; and Voice Onset Time of word-initial stops. This article also provides figures that exemplify individual speaker variation of vowel duration, F0, F1 and F2 as a function of focus conditions. The data can thus be potentially reused to observe individual variations in phonetic encoding of coda voicing contrast as a function of the aforementioned prosodically-conditioned factors (i.e., prosodic boundary, pitch accent, focus) in native vs. non-native English. Some theoretical aspects of the data are discussed in the full-length article entitled “Phonetic encoding of coda voicing contrast under different focus conditions in L1 vs. L2 English” [1].

**Specifications Table**

**Table:** 

Subject	Linguistics
Specific subject area	Phonetics, Second language acquisition
Type of data	Table Figure CSV file (Spreadsheet)
How the data were acquired	Acoustic measurements based on speech recorded in a laboratory setting. For the speech recording, a Tascam HP-Ps digital recorder and a SHURE KSN44 microphone were used at a sampling rate of 44.1 kHz. Praat [2] was used for the acoustic measurements.
Data format	Raw
Description of data collection	Preparation of the data involved acquisition of acoustic data via laboratory speech recording and acoustic measurements of F0, F1, F2, and duration of vowels in CVC target words; and the Voice Onset Time of the word-initial consonants in the target words.
Data source location	Hanyang University, Seoul, Korea
Data accessibility	With the article Repository name: OSF Direct URL to data: https://osf.io/fbms4 **DOI** 10.17605/OSF.IO/FBMS4
Related research article	J. Choi, S. Kim, T. Cho, Phonetic encoding of coda voicing contrast under different focus conditions in L1 and L2 English. *Front. Psychol*. 7: 624 (2016) https://doi.org/10.3389/fpsyg.2016.00624

## Value of the Data

• The data that are provided in a CSV formatted file contain acoustic measurements of English coda voicing, based on laboratory recordings of L1 and L2 groups: twelve native speakers of American English and twenty-four Korean learners of English. Half of the Korean speakers were advanced learners of English, and the other half were intermediate learners. The data is useful for researchers who aim to study various acoustic aspects of phonetic encoding of coda voicing contrast in different conditions that are related to prosodic structure in L1 and L2 English. They will also allow other researchers to examine effects of L2 proficiency.
• As the data were obtained from an equal number of female and male speakers in the two different language groups, researchers who are interested in individual and gender differences in phonetic encoding as well as L1 vs. L2 differences can benefit from these data.
• The data may also serve as a basis for further comparable experiments that are designed to examine how learners of L2 English with different native language backgrounds (other than Korean) phonetically manifest English coda voicing contrast in different prosodic conditions.

## 1 Objective

This dataset was prepared to be available to those researchers who are interested in investigating further how English coda voicing contrast is phonetically realized in terms of various phonetic correlates in L2 speech of non-native speakers of different L1 language backgrounds, in this case, Korean. In particular, in the original research article [1], the authors reported results based on vowel duration, F1 and F2 associated with the vowel production, but they did not report F0 data. So it remains to be seen how the coda voicing contrast in English produced by Korean learners of English is further reflected in the F0 correlate. Moreover, the dataset is expected to be used to examine L2 speaker variation, in particular, with respect to the Korean learners’ speaker-specific strategies in employing various phonetic correlates for encoding of the coda voicing contrast, and to what extent the L2 speaker variation is attributable to their native language background.

## 2 Data Description

### 2.1 A CSV file: acoustic measurements

A CSV file is available in the data repository (https://osf.io/fbms4). As exemplified in Table 1, the file contains the following information.

• The CSV file contains acoustic measurements of the vowel (V) and the preceding consonant in CVC target words produced by individual speakers.
• The “speaker_ID” column shows the codes used to identify participating speakers. “NAE” stands for native English speakers, “NK-Adv” stands for native Korean advanced learners of English, and “NK-Int” native Korean intermediate learners. The following two digits indicate the random number given to the speakers, which range from 01 to 12 for each speaker group. In the ‘speaker-gender’ column, “F” and “M” stand for female and male, respectively. The “group” column shows three speaker groups: “ENG-Nat”, “KOR-Adv”, and “KOR-Int”, each of which stands for English native speaker group, and native Korean speaker groups who are advanced vs intermediate learners of English. There are two conditions in the “native_lg” column: ENG for native speakers of English and KOR for native speakers of Korean.
• The “boundary” column indicates a prosodic boundary at which each test word occurs—i.e., “IP” for a target word occurring in an Intonational Phrase initial position and “Wd” for a target word occurring in an Intonation Phrase medial position. The reader is referred to speech materials in Section 2.2. There are three levels in the “focus-type”—i.e., “PH-FOC”, “LEX-FOC”, and “NoFoc”, standing for phonological focus, lexical focus, and no focus, respectively.
• The item column shows eight target words: *bet, bed, pet, ped, bat, bad, pat*, and *pad*. The onset column shows the onset consonant of the target words, which is either “p” or “b”. The “vowel_type” column shows vowels in target words, which are an English mid front vowel /ε/ or a low front vowel /æ/. The “coda_voicing” column shows the voicing of the coda consonant, which is either voiceless (for /t/) and voiced (for /d/). The “repetition” column is the number of repetitions of each carrier sentence containing a target word, ranging from 1 to 3.
• The remaining five columns show acoustic measurements. “F0_midpoint” indicates measured F0 values (Hz) taken at the midpoint of the vowel in CVC. “Vowel_duration” indicates duration of vowels (in ms) and “vot_duration” shows Voice Onset Time values (in ms) of the first (onset) consonant in CVC. Finally, “F1Hz” and “F2Hz” show the first and the second formant values of each vowel in Hertz, measured in the midpoint of the vowel. The following table illustrates the organization of the file.

**Table 1:** Part of the CSV file (Coda Voicing_L1ENG_L2KOR_RawData) for the purpose of illustration. The sample contains the acoustic measurement values for the target word *bad* as spoken by a native speaker of English (NAE01).

speaker ID	speaker _gender	group	native_lg	boundary	accent	focus _type	item	onset	vowel _type	coda _voicing	rep	F0_midpoint	vowel _duration	vot _duration	F1Hz	F2Hz
NAE01	F	ENG_Nat	ENG	IP	A	PH-FOC	BAD	B	Low	Voiced	2	253	251.7	6.6	994	2023
NAE01	F	ENG_Nat	ENG	IP	A	PH-FOC	BAD	B	Low	Voiced	3	239	219.4	5.5	1054	2122
NAE01	F	ENG_Nat	ENG	IP	A	PH-FOC	BAD	B	Low	Voiced	1	110	238.2	7.0	948	2078
NAE01	F	ENG_Nat	ENG	IP	A	LEX-FOC	BAD	B	Low	Voiced	2	244	235.4	6.0	1013	1989
NAE01	F	ENG_Nat	ENG	IP	A	LEX-FOC	BAD	B	Low	Voiced	3	231	235.3	5.6	1026	2058
NAE01	F	ENG_Nat	ENG	IP	A	LEX-FOC	BAD	B	Low	Voiced	1	231	258.0	8.7	973	2000
NAE01	F	ENG_Nat	ENG	IP	U	NoFOC	BAD	B	Low	Voiced	2	221	157.3	14.5	849	2099
NAE01	F	ENG_Nat	ENG	IP	U	NoFOC	BAD	B	Low	Voiced	3	204	145.2	6.6	957	2019
NAE01	F	ENG_Nat	ENG	IP	U	NoFOC	BAD	B	Low	Voiced	1	226	149.1	7.1	897	1965
NAE01	F	ENG_Nat	ENG	Wd	A	PH-FOC	BAD	B	Low	Voiced	2	221	238.8	6.3	1078	2045
NAE01	F	ENG_Nat	ENG	Wd	A	PH-FOC	BAD	B	Low	Voiced	3	193	254.9	7.6	1059	2153
NAE01	F	ENG_Nat	ENG	Wd	A	PH-FOC	BAD	B	Low	Voiced	1	204	205.0	6.0	952	2044
NAE01	F	ENG_Nat	ENG	Wd	A	LEX-FOC	BAD	B	Low	Voiced	2	205	246.0	6.4	1017	2070
NAE01	F	ENG_Nat	ENG	Wd	A	LEX-FOC	BAD	B	Low	Voiced	3	204	247.2	6.0	1045	2063
NAE01	F	ENG_Nat	ENG	Wd	A	LEX-FOC	BAD	B	Low	Voiced	1	200	268.7	9.2	962	2169
NAE01	F	ENG_Nat	ENG	Wd	U	NoFOC	BAD	B	Low	Voiced	2	193	120.3	11.7	888	2070
NAE01	F	ENG_Nat	ENG	Wd	U	NoFOC	BAD	B	Low	Voiced	3	93	134.9	0.0	883	2006
NAE01	F	ENG_Nat	ENG	Wd	U	NoFOC	BAD	B	Low	Voiced	1	187	115.6	13.6	881	2012

### 2.2 Figures

**Fig. 1:**
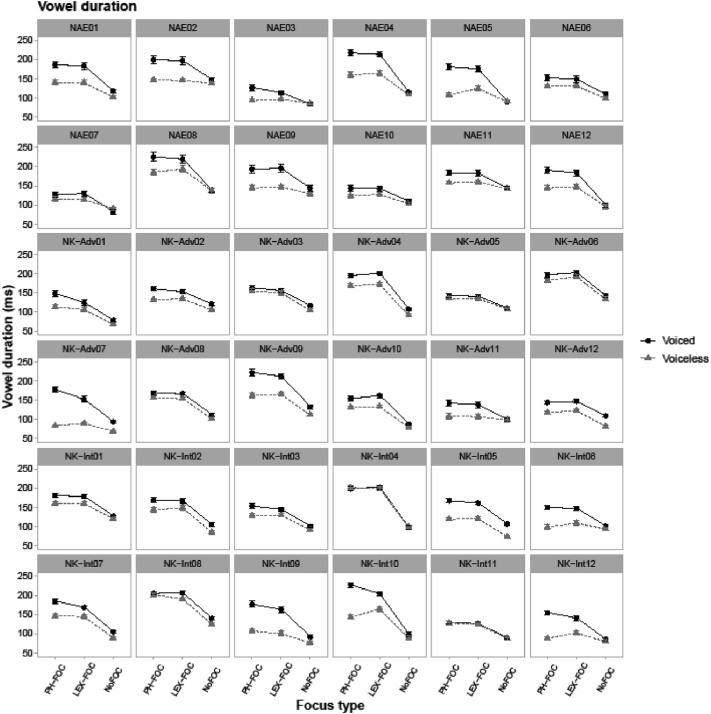
Line-point plots for 36 individual speakers showing each target word's vowel duration in ms (i.e., the duration of V in CVC target words). NAE stands for native American English speakers, NK-Adv stands for native Korean advanced learners of English, and NK-Int stands for native Korean intermediate learners of English. “PH-FOC”, “LEX-FOC”, and “NoFOC” indicate three focus conditions:phonological focus, lexical focus, and no focus. ‘Voiced’ (black) line points show patterns of target words with a voiced coda (e.g., *pad*) and ‘voiceless’ (gray) line points show patterns of target words with a voiceless coda (e.g., *pat*).

**Fig. 2:**
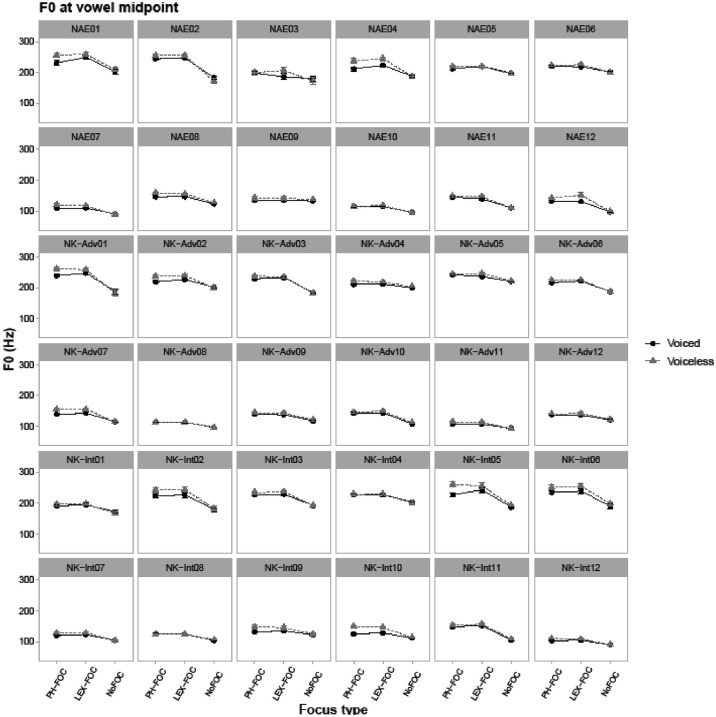
Line-point plots for 36 individual speakers showing vowels’ F0 values in Hz in CVC target words, taken at the midpoint of the vowel. NAE stands for native American English speakers, NK-Adv stands for native Korean advanced learners of English, and NK-Int stands for native Korean intermediate learners of English. “PH-FOC”, “LEX-FOC”, and “NoFOC” indicate three focus conditions: phonological focus, lexical focus, and no focus. ‘Voiced’ (black) line points show patterns of target words with a voiced coda (e.g., *pad*) and ‘voiceless’ (gray) line points show patterns of target words with a voiceless coda (e.g., *pat*).

**Fig. 3:**
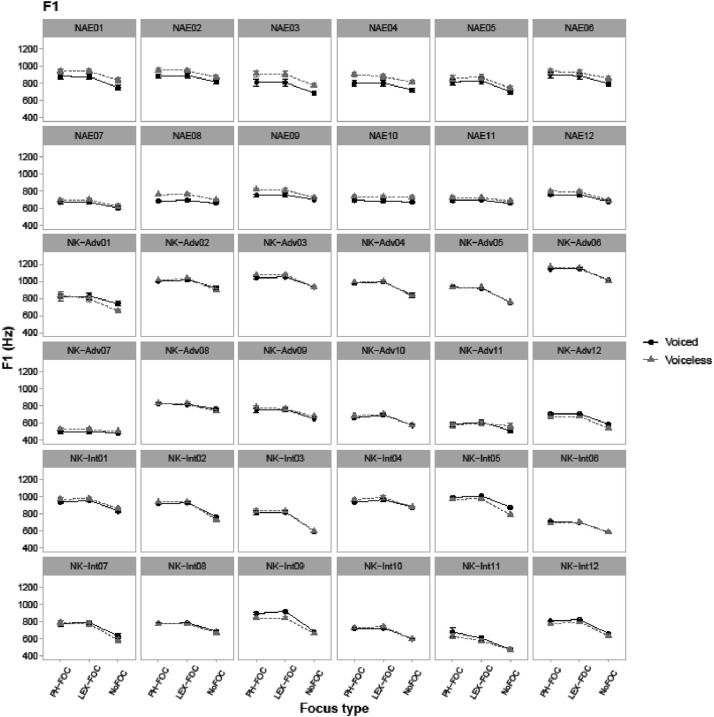
Line-point plots for 36 individual speakers showing vowel F1 values in Hz in the CVC target words, taken at the midpoint of the vowel. NAE stands for native American English speakers, NK-Adv stands for native Korean advanced learners of English, and NK-Int stands for native Korean intermediate learners of English. “PH-FOC”, “LEX-FOC”, and “NoFOC” indicate three focus conditions: phonological focus, lexical focus, and no focus. ‘Voiced’ (black) line points show patterns of target words with a voiced coda (e.g., *pad*) and ‘voiceless’ (gray) line points show patterns of target words with a voiceless coda (e.g., *pat*).

**Fig. 4:**
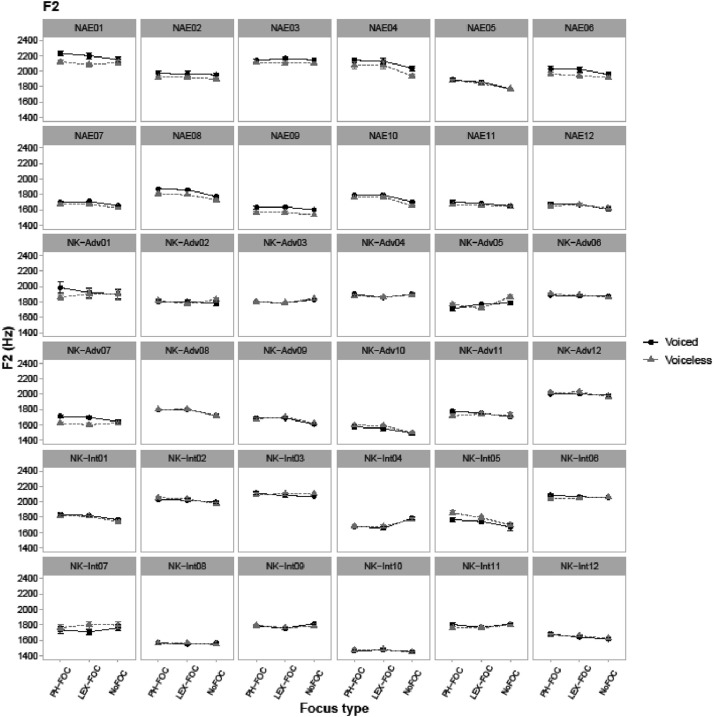
Line-point plots showing vowel F2 values in Hz in the CVC target words, measured at the midpoint of a vowel. Data are obtained from 36 speakers. NAE stands for native American English speakers, NK-Adv stands for native Korean advanced learners of English, and NK-Int stands for native Korean intermediate learners of English. “PH-FOC”, “LEX-FOC”, and “NoFOC” indicate three focus conditions of phonological focus, lexical focus, and no focus. ‘Voiced’ (black) line points show patterns of target words with a voiced coda (e.g., *pad*) and ‘voiceless’ (gray) line points show patterns of target words with a voiceless coda (e.g., *pat*).

**Fig. 5:**
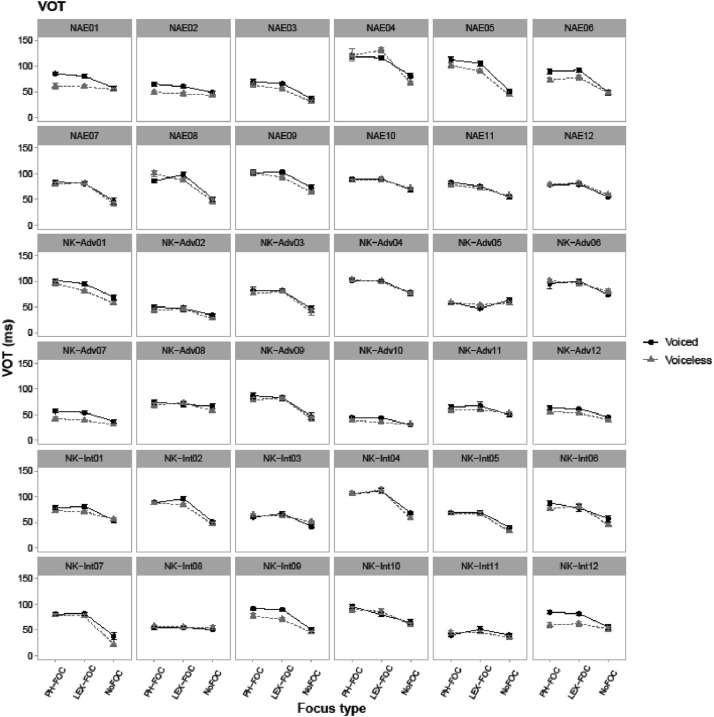
Line-point plots for 36 individual speakers showing Voice Onset Time (VOT) values in ms of the onset /p/ consonant in CVC target words of *pat, pad, pet*, and *ped*. NAE stands for native American English speakers, NK-Adv stands for native Korean advanced learners of English, and NK-Int stands for native Korean intermediate learners of English. “PHpH-FOC”, “LEX-FOC”, and “NoFOC” indicate three focus conditions of phonological focus, lexical focus, and no focus. ‘Voiced’ (black) line points show patterns of target words with a voiced coda (e.g., *pad*) and ‘voiceless’ (gray) line points show patterns of target words with a voiceless coda (e.g., *pat*).

Fig. 1, Fig. 2, Fig. 3, Fig. 4, Fig. 5 show individual speakers’ data (36 speakers) for phonetic encoding of coda voicing contrast as a function of focus type. Fig. 1 shows duration of the vowel in ms in CVC. Fig. 2, Fig. 3, Fig. 4 show measured values of F0, F1 and F2 in Hz, respectively, all of which were taken simultaneously at the midpoint of each vowel. Fig. 5 illustrates each speaker's Voice Onset Time (VOT) in ms for the word-initial /p/ consonant. Interested researchers can use the data included in the CSV file to obtain similar graphical illustrations as a function of other prosodic factors such as boundary and pitch accent.

## 3 Experimental Design, Materials and Methods

### 3.1 Participants

Participants were twelve native speakers of American English (Age range 21–33, Mean age 26), twelve native Korean advanced learners of English (Age range 21–26, Mean age 23), and twelve native Korean intermediate learners of English (Age range 21–28, Mean age 24). Within each group, a half of the participants was female, and the other half was male speakers. The English speakers were residing in Korea at the time of recording. All the Korean learners were college students at the time of recording. The division between advanced vs. intermediate learner groups was based on the learners’ TOEFL (Test of English as a Foreign Language) score. The advanced learners’ average TOEFL score was 110, and that of the intermediate learners was 75.

### 3.2 Speech Materials

The target words are four CVC minimal pairs in English which differ in terms of coda voicing and the vowel quality. The target (coda-contrasting) pairs with a mid front vowel are *bed*-*bet* and *ped*-*pet*; and those with a low front vowel are *bad*-*bat* and *pad*-*pat*.

The target words, as exemplified in Table 2, occur in a mini discourse situation as part of an answer to a given question. The mini discourse was designed in such a way to induce different focus types and prosodic boundaries. Table 2 shows example carrier sentences where “B” in each dialog is the carrier sentence which contains a target word *bed*.

**Table 2:** Example sentences with a target word *ped*. The target word is underlined and the focused words are in uppercase.

IP-initial (IP)	PH-FOC	A: Did you write ‘PET fast again’?
		B: Not exactly. ‘PED fast again’ was what I wrote.
	LEX-FOC	A: Did you write ‘CHAIN fast again’?
		B: Not exactly. ‘PED fast again’ was what I wrote.
	NoFOC	A: Did you write ‘ped SLOWLY again’?
		B: Not exactly. ‘ped FAST again’ was what I wrote.
IP-medial (Wd)	PH-FOC	A: Did you write ‘say PET fast’ again?
		B: No, I wrote ‘say PED fast’ again.
	LEX-FOC	A: Did you write ‘say CHAIN fast’ again?
		B: No, I wrote ‘say PED fast’ again.
	NoFOC	A: Did you write ‘say ped SLOWLY’ again?
		B: No, I wrote ‘say ped FAST’ again.

As illustrated in Table 2, the target words appear either at the beginning or in the medial of an Intonational Phrase (IP-initial vs IP-medial). In the phonological focus condition, the target word is contrasted in terms of the coda voicing with a word in the question “A” (e.g., PET vs. PED). Note that the term ‘phonological’ is used to refer to a phonemic contrast between voiced and voiceless coda consonants. In the lexical focus condition, the target word is contrasted ‘lexically’ with a semantically related word in the question “A” (e.g., CHAIN vs. PED). In the no focus condition, the corrective contrast fell on the word following a target word (e.g., SLOWLY vs. FAST) such that the target word was not the locus of focus.

The prompt questions in “A” were pre-recorded by a female native speaker of American English. During the recording, the participants listened to a prompt question and answered the question, based on the written dialog provided on the computer screen in front of them. The recording was conducted in a sound-proof booth, with a Tascam HP-Ps digital recorder and a SHURE KSN44 microphone at a sampling rate of 44.1 kHz.

### 3.3 Measurements

The acoustic measurements were taken using Praat [2]. For each target word CVC, the vowel duration was measured as an interval from the onset of F1 to the offset of F2. The values of F1, F2, and F0 were taken at the midpoint of the vowel. In addition, the Voice Onset Time of the onset (i.e., the first consonant in CVC) was measured from the point of the stop release to the onset of voicing for the vowel.

## Ethics Statements

The data collection did not involve experimentation, but it involved simple recording of the participants’ reading of speech materials (some written text). The data collection procedure followed general ethical protocols in accordance with the ethical requirements as described in the author guide for publication in Data in Brief. The study was reviewed and approved by the committee of the internal review board of HIPCS (Hanyang Institute for Phonetics and Cognitive Sciences of Language) in 2014. (Note that at the time of recording in 2014, the committee did not issue an approval number). Prior to the recording session, the participants had been informed that their participation in this non-experimental research was entirely on a voluntary basis; they could stop their participation at any time for any reason without any disadvantage; the data collection involved a recording of their read speech in front of a microphone in a sound attenuated recording booth; there were no known risks associated with the recording procedure; and the de-identified data would be studied. All these points were also written in the consent form. After they had been informed about all these points, the participants read the consent form again and signed it to participate in the research voluntarily.

## CRediT authorship contribution statement

**Sahyang Kim:** Conceptualization, Methodology, Investigation, Writing – original draft, Writing – review & editing, Funding acquisition. **Jiyoun Choi:** Methodology, Data curation, Investigation, Data curation, Visualization, Writing – original draft, Writing – review & editing. **Taehong Cho:** Conceptualization, Methodology, Investigation, Supervision, Writing – review & editing, Funding acquisition.

## Declaration of Competing Interest

The authors declare that they have no known competing financial interests or personal relationships that could have appeared to influence the work reported in this paper.

## Data Availability

Data of coda voicing contrast in English by native English and Korean speakers (Original data) (osf).
